# Ultraviolet Radiation Stimulates Activity of CO_2_ Concentrating Mechanisms in a Bloom-Forming Diatom Under Reduced CO_2_ Availability

**DOI:** 10.3389/fmicb.2021.651567

**Published:** 2021-03-16

**Authors:** Guang Gao, Wei Liu, Xin Zhao, Kunshan Gao

**Affiliations:** ^1^State Key Laboratory of Marine Environmental Science and College of Ocean and Earth Sciences, Xiamen University, Xiamen, China; ^2^Department of Technology and Resource Management, Guangdong Jiangmen Chinese White Dolphin Provincial Nature Reserve Management Bureau, Jiangmen, China

**Keywords:** bicarbonate uptake, carbonic anhydrase, microalgae, photochemical efficiency, photosynthesis, *Skeletonema costatum*

## Abstract

The diatom *Skeletonema costatum* is cosmopolitan and forms algal blooms in coastal waters, being exposed to varying levels of solar UV radiation (UVR) and reduced levels of carbon dioxide (CO_2_). While reduced CO_2_ availability is known to enhance CO_2_ concentrating mechanisms (CCMs) in this diatom and others, little is known on the effects of UV on microalgal CCMs, especially when CO_2_ levels fluctuate in coastal waters. Here, we show that *S. costatum* upregulated its CCMs in response to UVR (295–395 nm), especially to UVA (320–395 nm) in the presence and absence of photosynthetically active radiation (PAR). The intensity rise of UVA and/or UVR alone resulted in an increase of the activity of extracellular carbonic anhydrase (CAe); and the addition of UVA enhanced the activity of CCMs-related CAe by 23–27% when PAR levels were low. Such UV-stimulated CCMs activity was only significant at the reduced CO_2_ level (3.4 μmol L^−1^). In addition, UVA alone drove active HCO_3_^−^ uptake although it was not as obvious as CAe activity, another evidence for its role in enhancing CCMs activity. In parallel, the addition of UVA enhanced photosynthetic carbon fixation only at the lower CO_2_ level compared to PAR alone. In the absence of PAR, carbon fixation increased linearly with increased intensities of UVA or UVR regardless of the CO_2_ levels. These findings imply that during *S. costatum* blooming period when CO_2_ and PAR availability becomes lower, solar UVR (mainly UVA) helps to upregulate its CCMs and thus carbon fixation, enabling its success of frequent algal blooms.

## Introduction

Anthropogenically released chemicals such as chlorofluorocarbons (CFCs) and other chlorine-containing volatile gases have resulted in the decline of ozone in the stratosphere since the 1970s, leading to increasing UV radiation (UVR) on the ground (Stolarski, 1988; Staehelin et al., 2001; Manney et al., 2011). Due to the effective implementation of the Montreal Protocol, stratospheric ozone depletion has been stalled and a recovery in the Antarctic ozone hole seems to occur (Newman and Mckenzie, 2011; Solomon et al., 2016). However, in 2020, record-low ozone levels stretched across much of the central Arctic, covering an area about three times the size of Greenland (Witze, 2020). This vast ozone hole is probably the biggest on record in the Northern Hemisphere, which was caused by very chilly temperatures that induced the formation of high-altitude clouds and the ozone-destroying reactions. Therefore, variations of solar UVR reaching the earth surface remains uncertain. Solar radiation is essential for photosynthesis of primary producers on Earth, but its UV component (100–400 nm) could do harms to living organisms. UVR is commonly divided into three wavelength bands: UVA (315–400 nm), UVB (280–315 nm), and UVC (100–280 nm). Most UVA and partial UVB can reach the surface of Earth with UVC completely filtered out by ozone in the Earth’s atmosphere (mainly stratosphere). UVB only accounts for about 0.2% of total solar irradiance that reaches the ground at noon and UVA accounts for about 5% in the areas without ozone holes (Koronakis et al., 2002; Dias and von Sperling, 2017). It has been intensively reported that UVB can induce free oxygen radicals, impair the structure and function of DNA and proteins (Boelen et al., 2000; Gao et al., 2009), inhibit photosynthetic activity (Sobrino et al., 2008; Yin and Ulm, 2017), and thus reduce algal growth and primary productivity (Gao et al., 2009; Li et al., 2011; Domingues et al., 2017). On the other hand, UVA at low to moderate levels was found to stimulate carbon fixation of algae. For instance, UVA could stimulate photosynthetic carbon fixation of phytoplankton in coastal waters of South China Sea in summer by up to 25% (Li et al., 2011). The growth rate of brown alga *Fucus gardneri* embryos was also enhanced by UVA radiation (Henry and Van Alstyne, 2004). Previous studies about the effects of UVR usually employed different radiation treatments in the presence of PAR with or without UVR. Since there are potential interaction between UVR and PAR (Liu et al., 2014), it would be more essential to expose cells to UVR alone when investigating the effects of UVR. However, this kind of studies is very few. Gao et al. (2007) found that photosynthetic carbon fixation of phytoplankton assemblages in a coastal area (23°29'N, 117°06'E) of the South China Sea increased linearly with UVA or UVR alone. In addition, it was reported that UVA alone could be utilized by the red alga *Gracilaria lemaneiformis* to drive O_2_ evolution and carbon fixation (Xu and Gao, 2010; Xu et al., 2018). However, the potential mechanisms of UVR driving carbon fixation of algae remain unclear.

Carbon dioxide (CO_2_) is the essential substrate for algal photosynthesis. Although there are abundant dissolved inorganic carbon (DIC, ~2 mM) in seawater, the predominant form is HCO_3_^−^ with CO_2_ accounting for less than 1% (~13 μM). The key enzyme in photosynthetic carbon fixation, ribulose-1,5-bisphosphate carboxylase/oxygenase (Rubisco), that catalyzes the conversion of CO_2_ into organic carbon, has a relatively low affinity for CO_2_ and is commonly less than half saturated in most marine microalgae under current CO_2_ levels (Hopkinson et al., 2011; Goudet et al., 2020), limiting marine algal carbon fixation. Accordingly, most algae evolved CO_2_ concentrating mechanisms (CCMs), including enhanced carbonic anhydrase (CAe) activity, active uptake of HCO_3_^−^, acidified compartment, etc. (Giordano et al., 2005; Beardall and Raven, 2020), to deal with the CO_2_ limitation. Due mainly to human activities, CO_2_ level in seawater is predicted to increase in future (Pörtner et al., 2019) and, therefore, a large number of studies have been conducted to investigate the impacts of increased CO_2_ levels on algal CCMs and photosynthesis (Gao et al., 2017; Hennon et al., 2017; Raven et al., 2017; Porzio et al., 2018; Huang et al., 2019). On the other hand, CO_2_ level in coastal waters is very dynamic and could be lower than the current average value at noon when photosynthesis is intensive (Duarte et al., 2013; Raven et al., 2020). For instance, the pH at noon could reach 8.9 in seawater of toosh Island (48.32°N, 124.74°W) located in the Eastern Pacific, leading to a low CO_2_ condition (Wootton et al., 2008). During algal blooms, seawater pH could be extremely high with CO_2_ availability being very low. It was reported that pH level in the surface waters of Mariager Fjord, Denmark, achieved 9.75 during an algal bloom (Hansen, 2002). Algal tolerance to high pH and low CO_2_ varies among species, which may be related to the capacity of their CCMs (Björk et al., 2004; Gao et al., 2018a).

In terms of the combined effects of UVR and CO_2_ on diatoms, most studies used elevated CO_2_ levels compared to ambient levels (Sobrino et al., 2008; Domingues et al., 2014; Gao et al., 2018c) and little is known regarding the interaction of reduced CO_2_ levels and UVR. *Skeletonema costatum*, as a ubiquitous diatom species, can be found from equatorial to polar areas. It has optional applications in lipid diet and biofuels (Berge et al., 1995; Gao et al., 2019a). Meanwhile, it is an opportunist that can grow very rapidly and lead to algal blooms in many coastal waters when the conditions are suitable (Shikata et al., 2008; Gao et al., 2019b; Zeng et al., 2019). It has reported that both UVA and UVB reduced photosynthetic carbon fixation of *S. costatum* grown under incident solar radiation (Gao et al., 2009). On the other hand, the extracellular CA activity and photosynthetic carbon fixation of *S. costatum* (Greville) Cleve could be stimulated by moderate levels of UVA or UVR when treated with visible radiation (Wu and Gao, 2009), implying a possibility that UVR may modulate CCMs in this diatom, which are closely related to activity of its extracellular CAe (Chen and Gao, 2003). It seems likely that *S. costatum* have developed a strategy to cope with UVR and reduced CO_2_ availability during its blooming. Here, we hypothesized that *S. costatum* may utilize UVR to stimulate its CCMs to deal with lower CO_2_ condition and maintain its photosynthesis when the light intensity is low. In this study, we cultured cells of *S. costatum* at two levels of CO_2_ and exposed them to different levels of UVR to test this hypothesis by monitoring the change of carbon fixation, effective photochemical efficiency, CAe activity, HCO_3_^−^ utilization, etc.

## Materials and Methods

### Culture Conditions and Experimental Design

The experimental algal species, *S. costatum* (Greville) Cleve (strain 2042), was obtained from the Institute of Oceanography, Chinese Academy of Sciences. Before the experiment, cells were grown in sterilized seawater enriched with f/2 medium with the pH of 8.20 in an intelligent incubator (GXZ-300C, Jiangnan, Ningbo, China). The temperature was set at 20°C and intensity of PAR was 200 μmol photons m^−2^ s^−1^ (12:12 light:dark cycle). Cells at the exponential growth phase were harvested by centrifugation at 5,000 *g* for 10 min and transferred to two conditions. One was ambient CO_2_ (12.8 μmol L^−1^) and lower pH (8.2), and the other was lowered CO_2_ (3.4 μmol L^−1^) and higher pH (8.7) that simulates the situation when algal blooms occur (Brussaard et al., 1996). Before cultured at these two conditions, the harvested cells were washed twice with fresh media with the target CO_2_ levels. Cells were cultured in cylindrical quartz tubes (55 mm in diameter and 160 mm in length) and exposed to five radiation treatments (PAR, PAR + UVA, PAR + UVA + UVB, UVA, and UVA + UVB) for 1 h. The treatments of UVA and UVA + UVB were set to verify whether UVR alone could affect physiological performance of cells. Different radiance treatments were achieved by following operation: (1) PAR (395–700 nm), quartz tubes covered with Ultraphan film 395 (UV Opak; Digefra); (2) PAR + UVA (320–700 nm), quartz tubes covered with Folex 320 (Montagefolie no. 10155099; Folex, Dreieich, Germany); (3) PAR + UVA + UVB (295–700 nm), Ultraphan film 295 (UV Opak; Digefra, Munich, Germany); (4) UVA, a Folex 320 cutoff foil (Montagefolie, Nr. 10,155,099; Folex, Dreieich, Germany) was placed on the top of the PAR cutoff filter, UG11 (Schott, Mainz, Germany) to screen off UVB + UVC irradiance; and (5) UVA + UVB, a Ultraphan 295 foil (UV Opak; Digefra, Munich, Germany) was placed on the top of the UG11 filter (only UVR penetrable) to screen off UVC irradiance. Five levels of light intensity were set for five radiation treatments (Table 1), reflecting daily average light intensities of cloudy and sunny days in spring at Xiamen, China. Different radiance intensities were achieved by covering the tubes with different layers of neutral filtering screens. A solar simulator (Sol 1,200; Dr. Hönle GmbH, Martinsried, Germany) equipped with a 1,000-W xenon arc lamp was used for the experiment. The irradiances of PAR, UVA, and UVB were measured with a broadband filter radiometer (ELDONET, Real Time Computer, Möhrendorf, Germany). A cell concentration of 2 × 10^5^ cells ml^−1^ representative of the biomass density during algal blooms (Gao et al., 2019b) was inoculated and the changes of pH levels were within 0.01 units. Cultures were carried out in triplicate.

**Table 1:** Five radiation treatments and intensities (W m^−2^) were set in this study.

	PAR	PAR + UVA	PAR + UVA + UVB	UVA	UVA + UVB
Level 1	15.55	15.55 + 3.89	15.55 + 3.89 + 0.17	3.89	3.89 + 0.17
Level 2	30.24	30.24 + 7.56	30.24 + 7.56 + 0.33	7.56	7.56 + 0.33
Level 3	51.84	51.84 + 12.96	51.84 + 12.96 + 0.57	12.96	12.96 + 0.57
Level 4	93.31	93.31 + 23.33	93.31 + 23.33 + 1.02	23.33	23.33 + 1.02
Level 5	172.80	172.80 + 43.20	172.80 + 43.20 + 1.89	43.20	43.20 + 1.89

### Manipulation of Seawater Carbonate System

The two levels of pH (8.20 and 8.70) were obtained by adding NaHCO_3_ solution to sodium barbital buffered DIC-free seawater. DIC-free seawater was achieved by bubbling HCL acidified seawater (pH 2.0) with pure N_2_ for 1 h. The pH_NBS_ was measured using a pH meter (Orion STAR A211, Thermo Scientific, United States) that was calibrated with standard National Bureau of Standards (NBS) buffers (pH = 4.01, 7.00, and 10.01 at 25.0°C; Thermo Fisher Scientific Inc., United States). The DIC was measured using a total organic carbon analyzer (TOC-5000A; Shimadzu, Kyoto, Japan). CO_2_ level in seawater was calculated *via* CO2SYS (Pierrot et al., 2006) based on measured pH and DIC and known levels of nutrients, using the equilibrium constants of K1 and K2 for carbonic acid dissociation (Roy et al., 1993) and the KSO_4_^−^ dissociation constant from Dickson (1990).

### Measurement of Carbon Fixation

Photosynthetic carbon fixation was measured using a ^14^C method (Gao et al., 2009). Briefly, cells grown under different treatments were transferred to cylindrical quarts tubes (15 ml, 17 mm in diameter and 69 mm in length) and injected with 50 μl-2.5 μCi (0.0925 MBq) NaH^14^CO_3_ solution (ICN Radiochemicals, United Kingdom). The incubation was conducted for 1 h under five radiation treatments (PAR, PAR + UVA, PAR + UVA + UVB, UVA, and UVA + UVB). Three quartz tubes were wrapped with aluminum foil as dark control. After the incubations, cells were filtered onto Whatman GF/F glass filters and then transferred into 20 ml scintillation vials. The filters were fumed with a high concentration of HCl (12 mol L^−1^) in a fuming cupboard for12 h to expel the non-fixed inorganic carbon and then dried in an oven for 6 h at 45°C (Gao et al., 2007). Three milliliter of scintillation cocktail (Wallac Optiphase HiSafe 3, United States) was added to the vials and incubated for 2 h at room temperature. The ^14^C incorporated via photosynthesis was counted with a liquid scintillation counter (LS 6500, Beckman Coulter, Fullerton, CA, United States), and the carbon fixation rate was calculated by the following equation: carbon fixation rate [mg C (mg Chl *a*)^−1^ h^−1^] = [(CPM_(l)_-CPM_(d)_)/Ce] × I_f_ × DIC/A/T/C_Chl *a*_, where CPM_(l)_ and CPM_(d)_ are the numbers of counts per minute in the “light” bottle and in the “dark” bottle, respectively, Ce is the counting efficiency of the counter, If is the isotope discrimination factor, DIC is the concentration of total dissolved inorganic carbon, A is the number of added μCi added multiplied 2.2 × 10^6^, T is the incubation time, and C is the concentration of Chl *a* (Gao et al., 2007).

### Measurement of PSII Activity

Activity of PSII, presented by the effective photochemical efficiency, was estimated by a portable pulse amplitude modulated fluorometer (PAMWATER-ED, Walz, Effeltrich, Germany). After exposed to different radiation treatments for 1 h, samples were transferred to the cuvette of the fluorimeter. The measuring light and saturating pulse were 0.01 and 4,000 μmol photons m^−2^ s^−1^ (0.8 s), respectively. The actinic light pulse was set to the corresponding intensity of PAR in the corresponding radiation treatments (zero for UV-alone treatments). The effective photochemical efficiency was determined as: F'_v_/F'_m_ = (F'_m_ − F_t_)/F'_m_, where F'_m_ is the instant maximal fluorescence and F_t_ is the steady-state fluorescence of light-adapted cells.

### Estimate of Carbonic Anhydrase Activity

The activity of extracellular CAe of the intact cells was determined by an electrometric method based on Gao et al. (2009) with some modification. At the end of the exposure to different radiation treatments, the cells were immediately harvested by centrifugation (6,000 *g*, 4°C) for 10 min, washed and suspended in barbitone-buffered seawater (20 mM, pH 8.2). The cells were examined microscopically and no ruptured cells were found. One milliliter of cell suspension was added to 4 ml icy barbitone-buffered seawater and CO_2_-saturated icy distilled water (2 ml) was gently injected into the bottom of cell suspension (2 × 10^6^ cells/ml). The time required for the pH to decrease from pH 8.2 to 7.2 was recorded. During the reaction, cell suspension was stirred and the temperature was controlled at 4°C. Activity of the enzyme was calculated as follows: EU = 10 × (T_0_/T − 1), where T_0_ and T represent the time required for the pH change in the absence or presence of the cells, respectively.

### Test for pH Drift Under Different Radiation Treatments

To obtain the pH compensation point of *S. costatum* under different light conditions, cells grown at 200 μmol photons m^−2^ s^−1^ PAR were transferred to sealed quartz tubes (25 mm in diameter and 90 in length) and exposed to five radiation treatments at two levels of light intensity (Levels 3 and 5 in Table 1). The cell concentration for all treatments was 1.10 × 10^6^ cells/ml and the temperature was kept at 20°C. The change of medium pH was monitored with a pH meter (Orion STAR A211, Thermo Scientific, United States) and the pH compensation point was obtained when there was no further increase in pH.

### Statistical Analysis

Results were presented as means of replicates ± SD and data were analyzed with the software SPSS v.23. The data from each treatment conformed to a normal distribution (Shapiro-Wilk, *p* > 0.05) and the variances could be considered equal (Levene’s test, *p* > 0.05). Three-way ANOVA was conducted to assess the effects of CO_2_, radiation spectrum and intensity on carbon fixation, effective photochemical efficiency, extracellular CAe activity, and pH compensation point. Least significant difference (LSD) was conducted for *post hoc* investigation. Repeated-measures ANOVA was conducted to assess the effect of incubation time on media pH in a closed system, with Bonferroni for *post hoc* investigation. The threshold value for determining statistical significance was *p* < 0.05.

## Results

**Figure 1:**
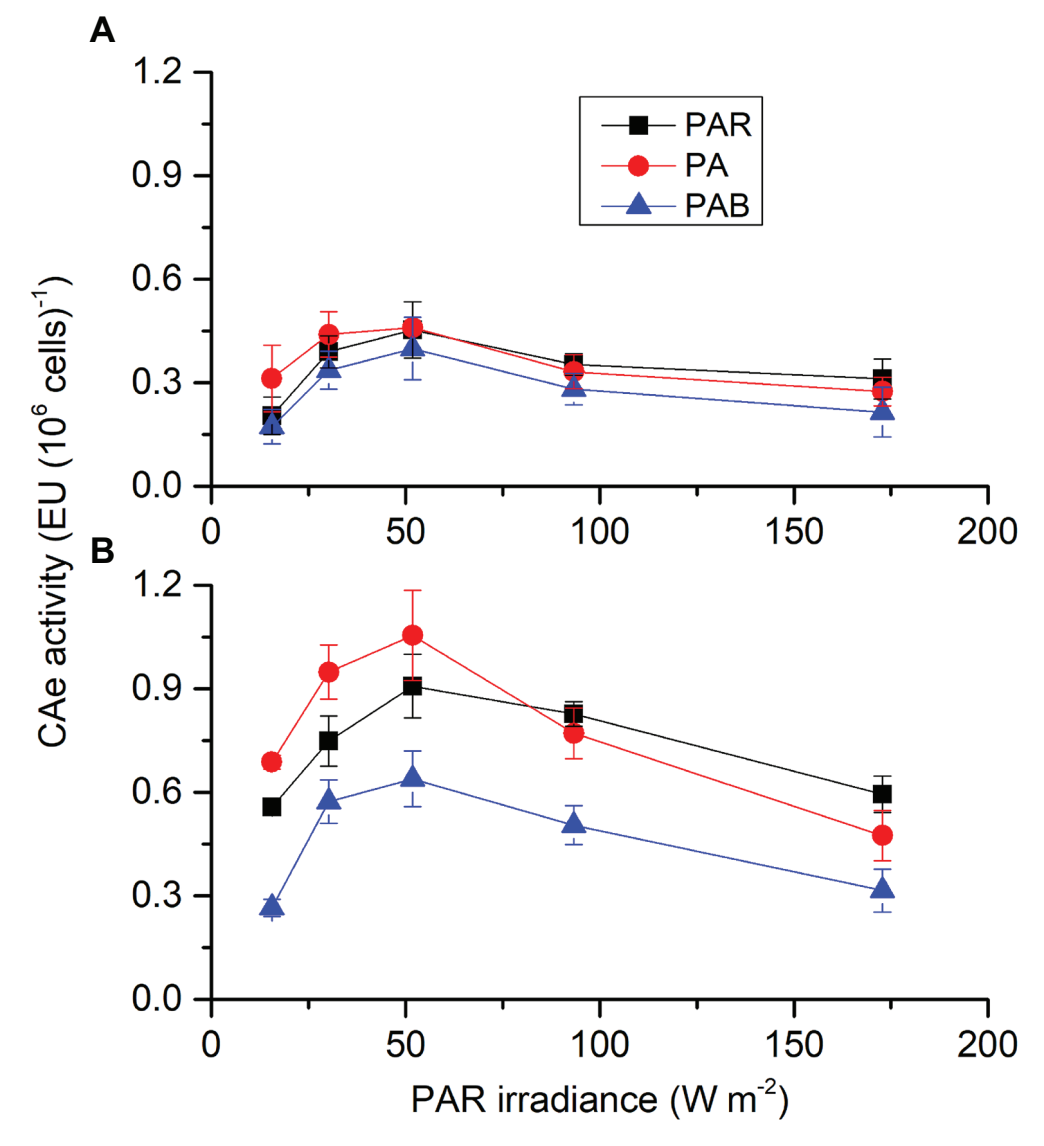
Activity of extracellular carbonic anhydrase (CAe) of *Skeletonema costatum* cultured at ambient **(A)** and lower CO_2_ **(B)** levels when exposed to different radiation treatments. PAR, photosynthetically active radiation; PA, PAR + UVA, PAB, PAR + UVA + UVB. The error bars indicate the SDs (*n* = 3).

Each factor (CO_2_, radiation spectrum, and intensity) affected CAe activity of *S. costatum* cells cultured in the presence of PAR and each two had an interactive effect (Supplementary Table S1). At ambient CO_2_ level (Figure 1A), CAe activity increased with radiation level until 51.84 W m^−2^ (238 μmol photons m^2^ s^−1^) PAR. There were no significant differences among radiation treatments. At lower CO_2_ level (Figure 1B), CAe activity increased first and then decreased with radiation level. Compared to PAR, PA stimulated CAe activity by 23 and 27% when PAR was 15.55 and 30.24 W m^−2^, respectively; PAB reduced it at all radiation levels. The lower CO_2_ level induced higher CAe activity at each treatment except for the highest PAB intensity.

**Figure 2:**
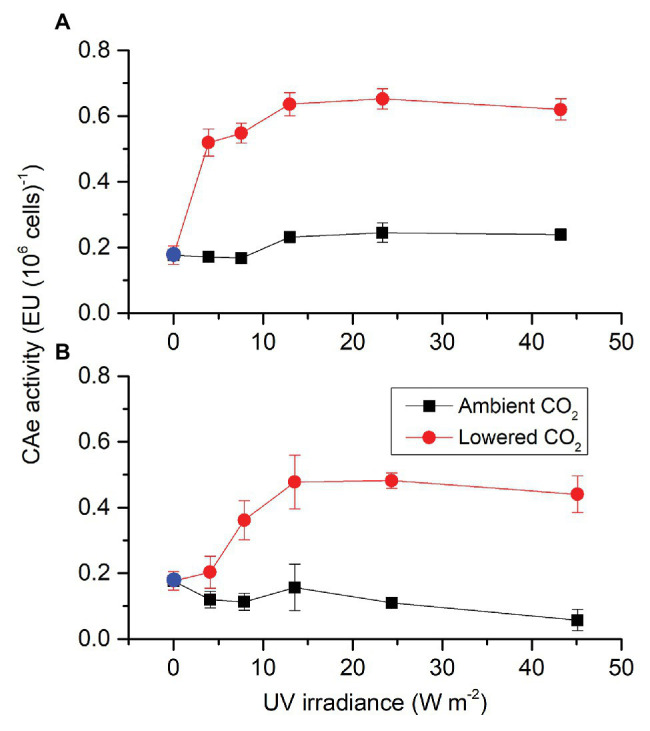
Activity of CAe of *S. costatum* grown at ambient and lowered CO_2_ levels when exposed to UVA **(A)** or UV radiation (UVR; UVA + UVB; **B**). The blue points represent the CA activity of cells cultured at pH 8.20 before exposed to UVR. The error bars indicate the SDs (*n* = 3).

Similar to CAe activity in the presence of PAR, each factor (CO_2_, radiation spectrum, and intensity) affected CAe activity of *S. costatum* cells cultured in the absence of PAR, each two had an interactive effect except for radiation spectrum and intensity, and these three also interacted (Supplementary Table S1). When cells were transferred from the growth condition to UVA exposure (Figure 2A), cells grown under lowered CO_2_ condition had a big leap in CAe activity and the CAe activity increased with UVA intensity until 12.96 W m^−2^. On the other hand, CAe activity of cells grown under ambient CO_2_ condition did not change when transferred from growth condition to UVA intensities below 12.96 W m^−2^ and afterward it increased to 0.23 ± 0.02 EU at 12.96 W m^−2^ and did not vary until the highest intensity of 43.20 W m^−2^. When cells were exposed to UVR (Figure 2B), CAe activity in cells grown at lowered CO_2_ also increased with UVR intensity until 13.53 W m^−2^. For cells grown at ambient CO_2_, CAe activity did not change with light intensity but the highest intensity reduced it compared to the lowest intensity. For both UVA and UVR exposure, cells grown at lowered CO_2_ had much higher CAe activity than those at ambient CO_2_.

**Figure 3:**
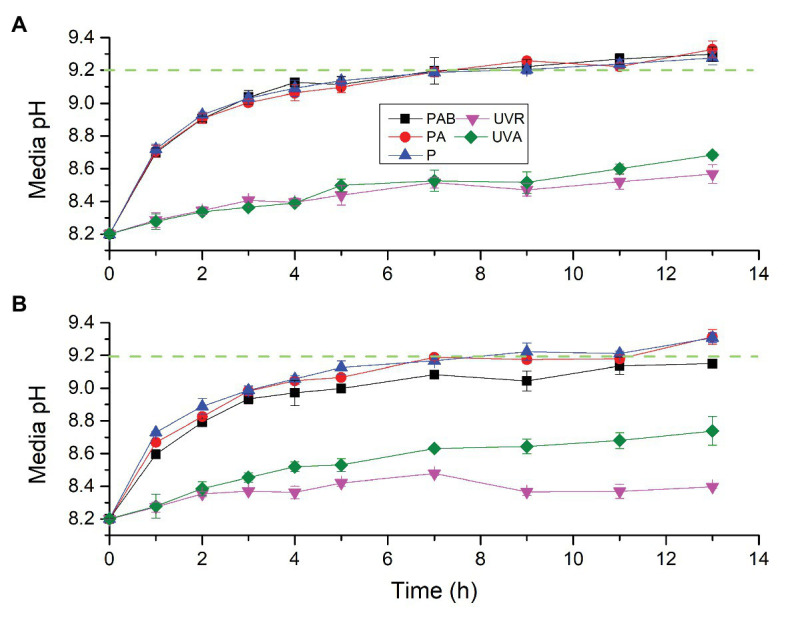
Changes of pH in a closed system driven by photosynthesis of *S. costatum* exposed to different light treatments. The initial pH was 8.20. The low **(A)** and high **(B)** density levels are 51.84 and 172.80 W m^−2^ for PAR, 12.96 and 43.20 W m^−2^ for UVA, and 13.53 and 45.09 W m^−2^ for UVR (UVA + UVB), respectively. The error bars indicate the SDs (*n* = 3). The dash line represents the pH compensation point above which indicates active HCO_3_^−^ uptake.

To test whether *S. costatum* can utilize HCO_3_^−^ even when exposed to UVR alone, pH drift experiment was conducted (Figure 3). Incubation time interacted with light intensity and spectrum to affect the pH in the diatom culture (Supplementary Table S2). At the low light intensity (Figure 3A), media pH under different light treatments increased with time. After 9 h exposure, the media pH under PAB, PA, and P exceeded 9.20, being 9.22 ± 0.05, 9.26 ± 0.01, and 9.20 ± 0.03, respectively. After 13 h exposure, media pH under PAB, PA, and P reached 9.30 ± 0.04, 9.33 ± 0.05, and 9.27 ± 0.04, respectively. No significant differences among PAB, PA, and P were found for each time point. During 13 h exposure, the media pH at UVA or UVA + UVB did not reach 9.20 and was largely lower than that with PAR. The media pH at UVA + UVB was lower than that at UVA at 11 and 13 h exposure but did not have differences at other time points.

At high light intensity (Figure 3B), media pH under different light treatments also increased with time. After 9 h exposure, only the media pH under PAR exceeded 9.20, being 9.22 ± 0.05, After 13 h exposure, the media pH under PAR and PA exceeded 9.20, being 9.31 ± 0.05 and 9.31 ± 0.03, respectively, while the media pH under PAB was still below 9.20, being 9.15 ± 0.02. The media pH under PAB was significantly lower than PAR since 5 h exposure. The media pH at UVR or UVA did not reach 9.20, being 8.40 ± 0.01 and 8.74 ± 0.09, respectively, after 13 h exposure. The media pH at UVR had been lower than that at UVA since 5 h exposure.

**Figure 4:**
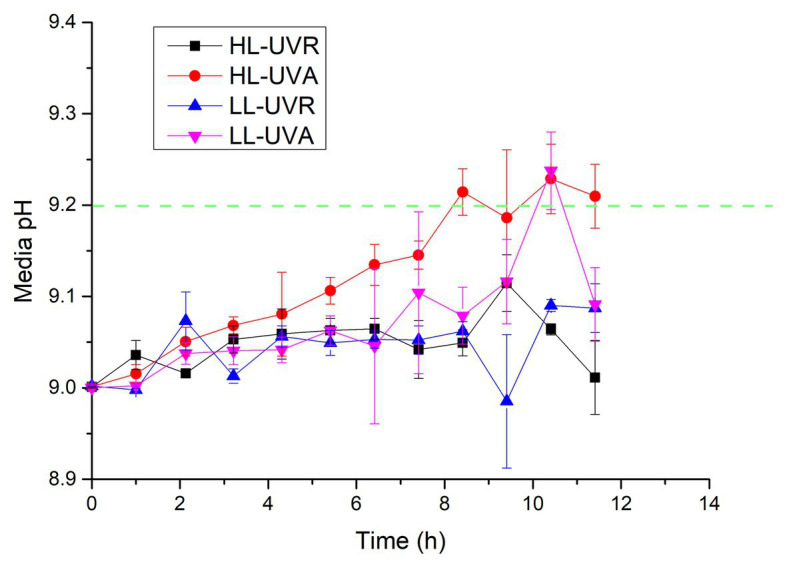
Changes of pH in a closed system driven by photosynthesis of *S. costatum* when exposed to different UVR treatments. The initial pH was 9.00. The low (LL) and high (HL) light densities are 12.96 and 43.20 W m^−2^ for UVA, and 13.53 and 45.09 W m^−2^ for UVR (UVA + UVB), respectively. The error bars indicate the SDs (*n* = 3). The dash line represents the pH compensation point above which indicates active HCO_3_^−^ uptake.

Although media pH at UVA or UVA + UVB did not reach 9.20 during 13 h exposure, they maintained a rising trend, indicating that the media pH may increase further if culture period is extended. Therefore, another pH drift experiment was conducted after setting the initial media pH 9.00 to verify whether *S. costatum* exposed to UVR only has the capacity of active uptake of HCO_3_^−^ (Figure 4). Exposure time interacted with light intensity and spectrum to affect media pH over 11.4 h of incubation (Supplementary Table S2). Media pH exposed to HL-UVA increased with exposure time and reached 9.21 ± 0.03 and 9.23 ± 0.04, respectively, after 8.4 and 10.4 h culture. Media pH exposed to LL-UVA also rose with exposure time and reached 9.23 ± 0.04 after 10.4 h culture. On the other hand, media pH exposed to UVR did not show significant increase during 11.4 h of incubation.

**Figure 5:**
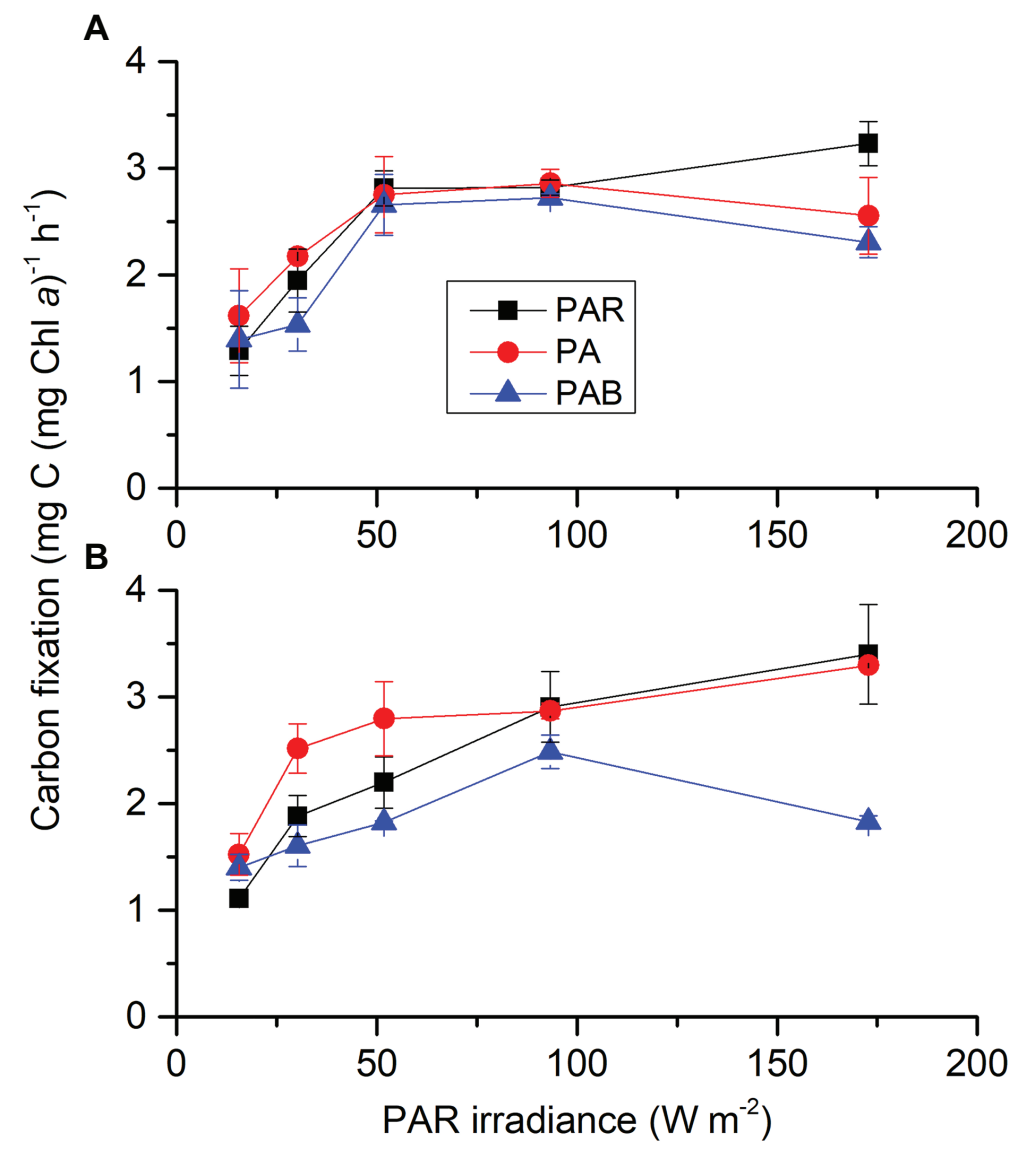
Photosynthetic carbon fixation rates of *S. costatum* grown at ambient **(A)** and lowered CO_2_ **(B)** levels when exposed to different radiation treatments. PAR, photosynthetically active radiation; PA, PAR + UVA, PAB, PAR + UVA + UVB. The error bars indicate the SDs (*n* = 3).

Carbon dioxide did not affect carbon fixation but both radiation spectrum and intensity affected it; each two factors had an interactive effect (Supplementary Table S3). At ambient CO_2_ (Figure 5A), presence of UVA (PAR + UVA) or UVR (PAR + UVA + UVB) did not affect carbon fixation at lower radiation levels (PAR ≤ 93.31 W m^−2^, 429 μmol photons m^2^ s^−1^) compared to PAR alone, though addition of UVA showed slightly higher values than that with UVR at PAR of 30.24 W m^−2^ (139 μmol photons m^2^ s^−1^). At higher radiation level [PAR = 172.80 W m^−2^ (795 μmol photons m^2^ s^−1^), UVA = 43.2 W m^−2^, and UVB = 1.89 W m^−2^], presence of UVA or UVR reduced carbon fixation by 20.35 and 28.66%, respectively. At lowered CO_2_ level (Figure 5B), the carbon fixation rate was higher with UVA compared to that under PAR alone at lower PAR levels (15.55 and 30.24 W m^−2^, corresponding to 72 and 139 μmol photons m^2^ s^−1^). However, UVA did not bring about such enhancement under higher PAR levels (PAR ≥ 51.84 W m^−2^, 238 μmol photons m^2^ s^−1^). Presence of UVR did not affect carbon fixation at lower radiation levels (PAR ≤ 93.31 W m^−2^, 429 μmol photons m^2^ s^−1^) but significantly reduced it at the highest radiation level (PAR = 172.80 W m^−2^, 795 μmol photons m^2^ s^−1^) by 46.18%.

**Figure 6:**
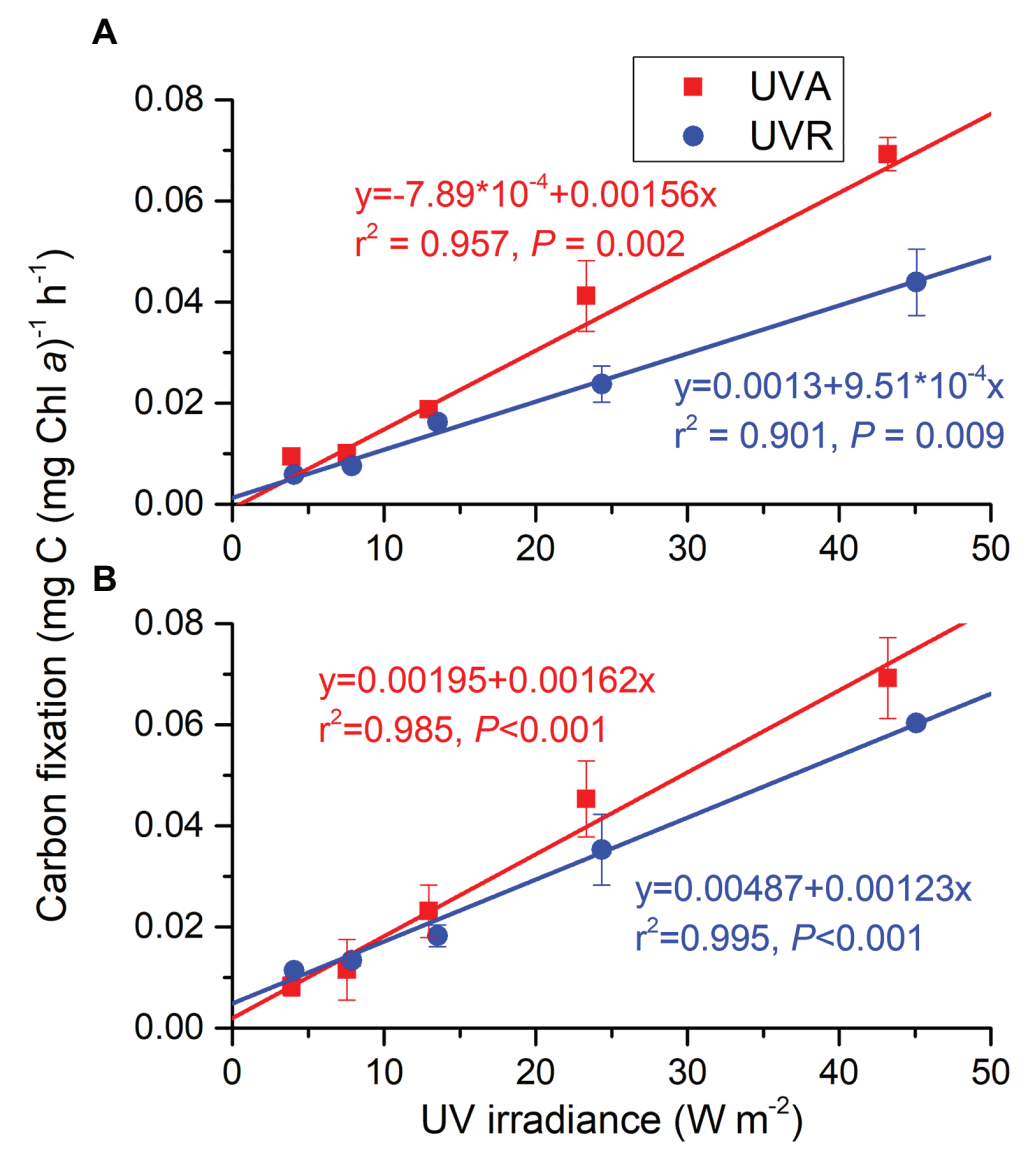
Carbon fixation rates of *S. costatum* grown at ambient **(A)** and lowered CO_2_ **(B)** levels when exposed to UVA or UVR (UVA + UVB). The error bars indicate the SDs (*n* = 3).

Changed CO_2_ levels did not affect UVR-driving carbon fixation; UVR spectrum and intensity affected it and they had an interactive effect (Supplementary Table S3). At ambient CO_2_ level (Figure 6A), carbon fixation of the cells grown under UVA or UVR alone increased linearly with radiation level (R^2^ = 0.957, *p* = 0.002 for UVA; R^2^ = 0.901, *p* = 0.009 for UVR), with the rate being higher under UVA than UVR at each radiation level, reflecting an inhibitory impacts of UVB. The gap between UVA and UVR became larger with increasing radiation levels. At the lowered CO_2_ level (Figure 6B), carbon fixation of cells grown under both UVA and UVR increased as well with increasing radiation levels (R^2^ = 0.985, *p* < 0.001 for UVA; R^2^ = 0.995, *p* < 0.001 for UVA + UVB), but the difference between UVA and UVR was not significantly different at any radiation level. For UVA, there was no significant difference between two CO_2_ levels at any radiation level. For UVR, ambient CO_2_ reduced carbon fixation although the decrease at 13.527 or 24.35 W m^−2^ was not statistically significant.

**Figure 7:**
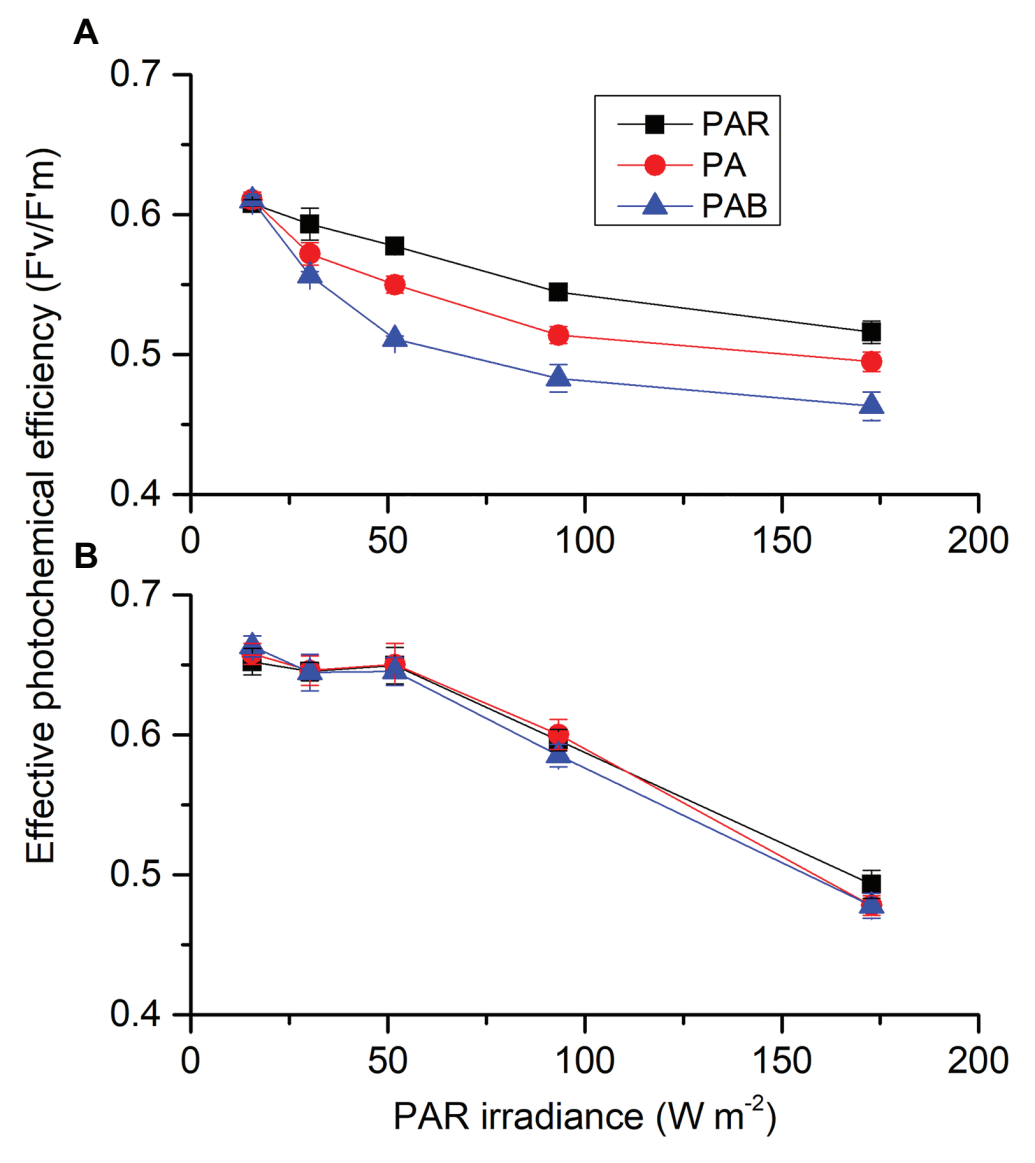
Effective photochemical efficiency (F'_v_/F'_m_) of *S. costatum* cultured at ambient **(A)** and lower CO_2_ **(B)** levels exposed to different radiation treatments. PAR, photosynthetically active radiation; PA, PAR + UVA, PAB, PAR + UVA + UVB. The error bars indicate the SDs (*n* = 3).

Each factor (CO_2_, radiation spectrum, and intensity) affected PAR-driving F'_v_/F'_m_, each two had an interactive effect and these three also interacted (Supplementary Table S4). At ambient CO_2_ level (Figure 7A), F'_v_/F'_m_ decreased with radiation level. When PAR level was above 30.24 W m^−2^ (139 μmol m^2^ s^−1^), PA reduced F'_v_/F'_m_ and PAB led to a larger decrease compared to PAR. At lowered CO_2_ level (Figure 7B), F'_v_/F'_m_ also decreased with radiation level, but there were no differences among three radiation treatments (PAR, PA, and PAB). For each radiation treatment, cells had higher F'_v_/F'_m_ at lower CO_2_ compared to ambient CO_2_ except for the highest PAB level.

**Figure 8:**
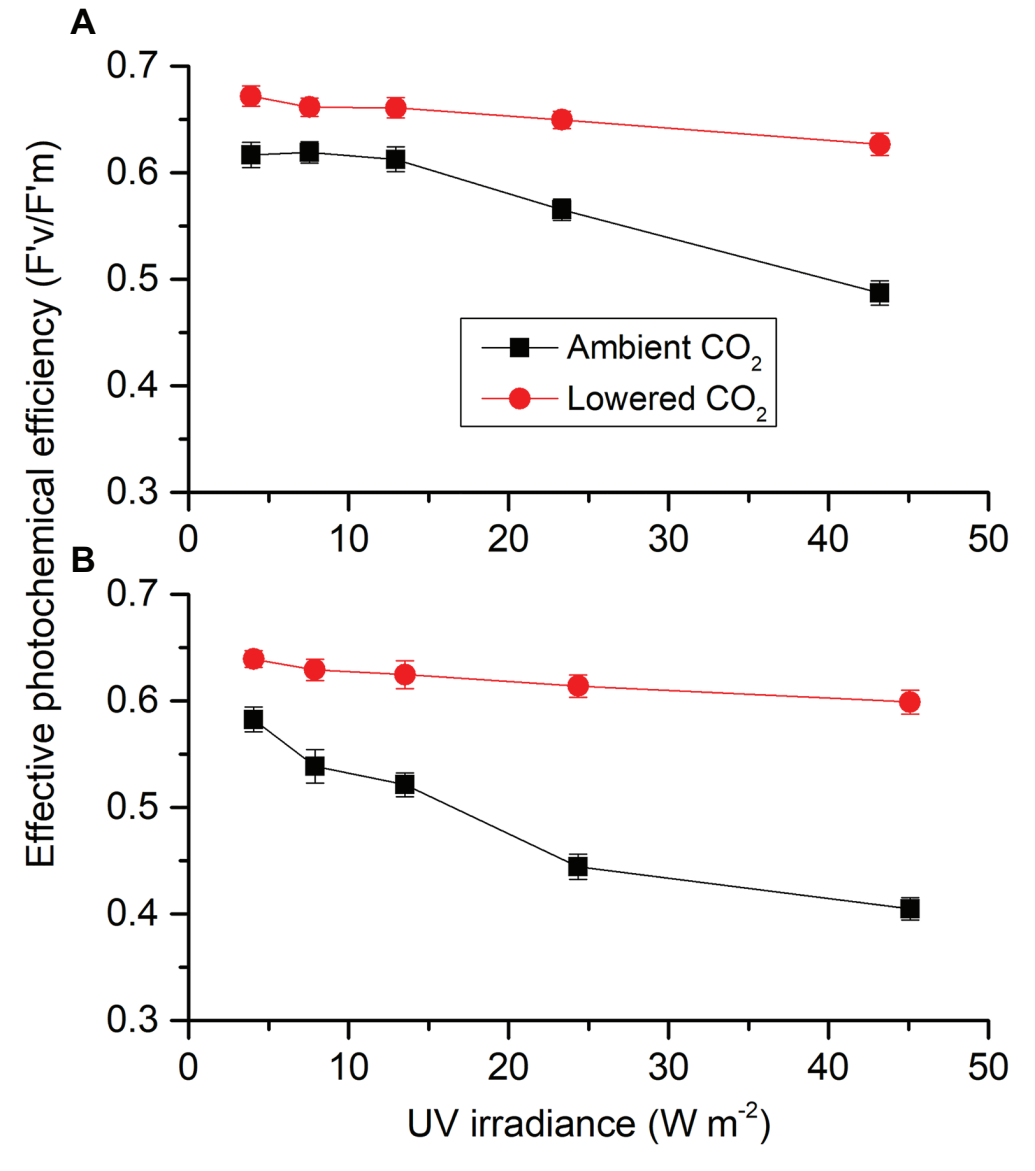
Effective photochemical efficiency (F'_v_/F'_m_) of *S. costatum* cultured at ambient and lowered CO_2_ when exposed to UVA **(A)** or UVR (UVA + UVB; **B**). The error bars indicate the SDs (*n* = 3).

Similar to PAR-driving F'_v_/F'_m_, each factor (CO_2_, radiation spectrum, and intensity) affected UVR-driving F'_v_/F'_m_, each two had an interactive effect and these three also interacted (Supplementary Table S4). When cells were exposed to UVA (Figure 8A), F'_v_/F'_m_ did not change until UVA passed 12.96 W m^−2^ for both ambient and lowered CO_2_ conditions. Compared to the lowest intensity, the highest UVA intensity resulted in a decrease of 6.75% at lowered CO_2_ level while it was 20.97% for ambient CO_2_ level. When cells were exposed to UVR (Figure 8B), F'_v_/F'_m_ did not change until UVR passed 13.53 W m^−2^ for lower CO_2_ level but it began to decrease when the light intensity was above 7.89 W m^−2^ for ambient CO_2_ level. Compared to the lowest light intensity, the highest UVR intensity resulted in a decrease of 6.31% at lower CO_2_ level while it was 30.49% for ambient CO_2_ level. F'_v_/F'_m_ at lower CO_2_ was higher than that at ambient CO_2_ for each light intensity. For both ambient and lower CO_2_, cells had lower F'_v_/F'_m_ under UVR compared to those under UVA at each radiation level.

## Discussion

In the present work, we found that the diatom *S. costatum* up-regulated its CCMs due to synergistic effects of solar UVR and reduced CO_2_ availability, and showed that even UVR alone (without the presence of PAR) could stimulate the activity of CAe, induce active HCO_3_^−^ uptake and thus promote photosynthetic carbon fixation. These results showed the first evidence that UVR alone can stimulate CCMs-mediated carbon acquisition.

### UVR Enhancing CCMs Activity at Reduced CO_2_ Level

Carbonic anhydrase, which catalyzes the interconversion between CO_2_ and water and the dissociated ions of carbonic acid, is deemed as an important component of algal CCMs (Giordano et al., 2005; Beardall and Raven, 2020). Therefore, changes in CA activity have been considered as a signal of regulation of CCMs. Lowered CO_2_ availability usually induces CA activity to different extents under different levels of nutrients (Gao et al., 2018a,b). While it is known that decreased CO_2_ concentration and increased levels of light can trigger higher CA activity in *S. costatum* (Chen and Gao, 2003; Giordano et al., 2005), only limited knowledge has been documented on effects of UVR on it (Wu and Gao, 2009). In this work, the addition of UVA to PAR enhanced CAe activity, while UVB appeared to be inhibitive when combined with UVA. On the other hand, the CAe activity of cells grown at higher CO_2_ was not induced by UVA or UVR (Figure 1). This might be caused the downregulation of CCMs by the higher CO_2_ level, consequently dampening the UV-mediated effects on CCMs activity. Furthermore, we provided the first evidence that UVR alone drove CAe activity (Figure 2). This may be related to the change of the redox activity in the plasma membrane induced by UVA (Wu and Gao, 2009) and additionally the energy of UVA may also be utilized for the synthesis of CA through photosynthetic electron transport.

Active uptake of HCO_3_^−^ is another pathway of algal CCMs (Giordano et al., 2005). A pH compensation point over 9.2 has been deemed as a sign of active HCO_3_^−^ uptake for algae because there is hardly any CO_2_ available in water with a pH above 9.2 (Axelsson and Uusitalo, 1988; Gao et al., 2018a). In the present study, the addition of UVA to PAR promoted active uptake and utilization of HCO_3_^−^, so that pH increased to higher extent (Figure 3A). On the other hand, active uptake of HCO_3_^−^ did not occur when PAR + UVR increased to a higher level. Sobrino et al. (2005) speculated that UVR may damage one or more of the processes involved with HCO_3_^−^ transport in *Nannochloropsis gaditana*. Our pH drift tests showed that damage of UVR on algal HCO_3_^−^ transport was due to UVB rather than UVA, since the cells exposed to PAR + UVA maintained a high pH compensation point over 9.2 while addition of UVB led to lower value (Figure 3B), reducing the pH compensation point to the value below 9.2. In addition, UVA alone drove HCO_3_^−^ transport and utilization in *S. costatum* while UVR did not make it (Figure 4), indicating the damaging effect of UVB again. To the best of our knowledge, this is the first report on the UVR-driving active uptake of HCO_3_^−^ in algae. As shown in this study, UVR can drive electron transport in PSII and the generated ATP may be utilized for active uptake of HCO_3_^−^. This can help to explain why UVA alone can drive photosynthetic carbon fixation at low CO_2_ levels.

### UVR Driving Carbon Fixation Under Changing CO_2_ Levels

In this study, exposures to moderate levels of UVA or UVR in the presence of PAR did not affect carbon fixation of *S. costatum*, and high levels of UVR (UVA of 43.2 W m^−2^ or UVR of 45.1 W m^−2^) significantly reduced its carbon fixation under ambient CO_2_ level (Figure 5A). On the other hand, when CO_2_ level decreased to 2.8 μmol L^−1^, the addition of UVA to PAR increased carbon fixation of *S. costatum* grown at lower irradiance levels and did not reduce it even at the highest irradiance level (Figure 5B). The stimulating effect of UVA on carbon fixation was also found in phytoplankton assemblages in coastal waters of the South China Sea (Gao et al., 2007; Li et al., 2011). Such stimulation is most likely due to compounded effects of the UV-mediated CCMs activity and photosynthetic capture of UVA as a supply of energy. Since the CCMs activity was only induced with reduced availability of CO_2_ in *S. costatum* (Chen and Gao, 2003), our data indicate that UVR, especially, UVA only functions positively during the period when CCMs regulation is modulated, and there is a threshold beyond which UVR turns to damage CCMs-related proteins and inhibit carbon fixation.

UVA or UVR alone drove carbon fixation of *S. costatum* with lesser extent in the presence of UVB, indicating its inhibitory effect (Figure 6), which is the first time evidenced in this diatom. While diatom-dominated phytoplankton assemblages in the coastal waters were shown to be capable of photosynthesizing, and presence of UVB exhibited inhibitory effects (Gao et al., 2007), our results support this by providing species-level evidence. In the present work, presence of UVB reduced carbon fixation at ambient CO_2_ level but did not significantly affect it at lower CO_2_ level, suggesting that low to moderate levels of UVB may be utilized under lower CO_2_ level. Such a utilization of UVB has also been reported in a freshwater cyanobacterium *Nostoc sphaeroides*, in which photosynthesis and biomass production were even enhanced by low-dose UVB *via* enhancing the primary quinone-type acceptor (QA) re-oxidation, plastoquinone (PQ) pool re-oxidation, photosystem I (PSI) content, and cyclic electron transfer around PSI (Chen et al., 2020). Subsequently, both UVA and UVB may play roles in signaling for additional processes in addition to photosynthetic carbon fixation and CCMs.

### UVR Driving Light Reaction Under Changing CO_2_ Levels

The addition of UVA or UVR to PAR significantly reduced the effective photochemical efficiency (F'_v_/F'_m_) of *S. costatum* grown at ambient CO_2_ (Figure 7A). Similar results were also found in the diatoms *Cylindrotheca closterium* f. *minutissima* (Wu et al., 2012) and *Thalassiosira weissflogii* (Gao et al., 2018c). It has been documented that UVR could inhibit PSII activity by impairing photosynthetic apparatus, such as PSII subunits PsbA (D1), and PsbD (D2) proteins (Six et al., 2007; Gao et al., 2018c). In contrast, the addition of UVA or UVR to PAR did not affect F'_v_/F'_m_ under lowered CO_2_ condition even at the highest irradiance levels (Figure 7B). The energy of UV should be consumed during low CO_2_-induced upregulation of CCMs and hence the PSII was protected. Compared to lower CO_2_ (13.3 μmol L^−1^), higher CO_2_ (33.7 μmol L^−1^) exacerbated the stress of UVR on photosystem II function in the diatom *T. weissflogii* through reducing PsbD removal rate and the ratio of RbcL to PsbA during UVR exposure (Gao et al., 2018c). In the present study, compared to the cells grown at 2.8 μmol L^−1^ CO_2_, those grown at 10 μmol L^−1^ CO_2_ were more vulnerable to UVR in terms of photochemical performance, combined with the study by Gao et al. (2018c) study, indicating that the sensitivity of diatom to UVR may increase with the increase of CO_2_ level; therefore, inducing higher NPQ was in induced as reported in another work (Li et al., 2012). Under reduced CO_2_ availability, light energy could be transported and converted quickly and thus UVR did not reduce activity of PSII, which could be due to the upregulation of CCMs (Gao et al., 2012). Under lower CO_2_ conditions, intracellular CO_2_ is not enough to saturate carbon fixation. CCMs are usually activated or upregulated to increase intracellular DIC pools but the operation of CCMs is energy-consuming (Mishra et al., 2018). Since UVR can be used to stimulate CCMs in *S. constatum*, it could be quickly drained to fuel the energy demand associated with CCMs, avoiding the harm to PSII. Furthermore, UVA or UVR alone could drive electron transport in PSII (Figure 8). This indicates that the carbon fixation driven by UVA or UVR was through the electron transport and operation of PSII that supplies ATP and NADPH, following the same pathway as PAR.

## Conclusion

The findings in the present study verifies the hypothesis proposed in the introduction that *S. costatum* could utilize UVR particularly UVA to drive carbon fixation at the lower CO_2_ condition by stimulating CCMs. The only deviated result is that presence of UVB, when combined with UVA, did not stimulate the activity of CCMs, carbon fixation, or photochemical quantum yield. Therefore, it is shorter wavebands of UVB that may have played an inhibitory role. The photons of UVA can be absorbed and transported through PSII and transformed into chemical energy ATP, which can be used to upregulate CCMs via enhancing CA activity and HCO_3_^−^ utilization. Such UV-utilizing capability is particularly important for *S. costatum* to maintain survival and growth when PAR is low. With increased biomass density during the diatom blooms, both CO_2_, light, and UV levels are usually reduced due to self-shading. Therefore, harmful impacts of high levels of UVR can hardly be expected; instead, the low levels of UVR can promote CCMs and thus carbon fixation, so that successful and frequent occurrence of diatom blooms becomes possible. More studies in future should be conducted to investigate whether other diatoms or algae could also utilize UVR to upregulate CCMs when CO_2_ and PAR are in low levels.

## Data Availability Statement

The raw data supporting the conclusions of this article will be made available by the authors, without undue reservation.

## Author Contributions

GG: conceptualization, investigation, methodology, formal analysis, visualization, writing – original draft preparation, writing – reviewing and editing, and funding acquisition. WL: writing – reviewing and editing, investigation, and methodology. XZ: methodology, investigation, and writing – reviewing and editing. KG: conceptualization, methodology, writing – original draft preparation, writing – reviewing and editing, supervision, and funding acquisition. All authors contributed to the article and approved the submitted version.

### Conflict of Interest

The authors declare that the research was conducted in the absence of any commercial or financial relationships that could be construed as a potential conflict of interest.

The handling editor declared a past co-authorship with one of the authors [KG].
