# Supplementary material for: Ultraviolet Radiation Stimulates Activity of CO2 Concentrating Mechanisms in a Bloom-Forming Diatom Under Reduced CO2 Availability
Source: Front Microbiol. 2021 Mar 16;12:651567. doi: 10.3389/fmicb.2021.651567 (PMC8008072; doi:10.3389/fmicb.2021.651567)
Supplement: Supplementary file 1 [file Data_Sheet_1.docx]

**Ultraviolet radiation stimulates activity of CO_2_ concentrating mechanisms in a bloom-forming diatom under reduced CO_2_ availability**

Guang Gao^1^, Wei Liu^2^, Xin Zhao^1^, Kunshan Gao^1^*

^1^State Key Laboratory of Marine Environmental Science and College of Ocean and Earth Sciences, Xiamen University, Xiamen 361005, China

^2^Department of Technology and Resource Management, Guangdong Jiangmen Chinese White Dolphin Provincial Nature Reserve Management Bureau, Jiangmen 529000, China

* Correspondence:

Kunshan Gao

ksgao@xmu.edu.cn

**Table S1.** Three-way analysis of variance for the effects of CO_2_, radiation spectrum, and radiation intensity on PAR- and UVR-driving CAe activity of *S. costatum.* The asterisk means the interactive effect, df means degree of freedom, F means the value of F statistic, and Sig. means *p*-value.

| Source | PAR-driving CAe activity | | | UVR-driving CAe activity | | |
| --- | --- | --- | --- | --- | --- | --- |
|  | df | F | Sig. | df | F | Sig. |
| CO_2_ | 4 | 90.952 | <0.001 | 4 | 189.003 | <0.001 |
| Spectrum | 5 | 21.520 | <0.001 | 4 | 43.992 | <0.001 |
| Intensity | 4 | 49.049 | <0.001 | 4 | 17.730 | <0.001 |
| CO_2_* Spectrum | 8 | 7.207 | <0.001 | 4 | 7.349 | <0.001 |
| CO_2_*Intensity | 4 | 7.273 | <0.001 | 4 | 8.011 | <0.001 |
| Spectrum*Intensity | 8 | 3.0804 | 0.007 | 4 | 1.968 | 0.124 |
| CO_2_*Spectrum* Intensity | 8 | 1.181 | 0.330 | 4 | 5.957 | 0.001 |
| Error | 48 |  |  | 31 |  |  |

**Table S2.** Repeated measures analysis of variance for the effects of light intensity and spectrum on pH change with 13 hours of incubation. Time*Intensity means the interactive effect of incubation time and light intensity, Time*spectrum means the interactive effect of incubation time and light spectrum, Time*Intensity*Spectrum means the interactive effect of incubation time, light intensity and spectrum, df means degree of freedom, F means the value of F statistic, and Sig. means p-value.

| Source | Media pH starting from 8.20 | | | Media pH starting from 9.00 | | |
| --- | --- | --- | --- | --- | --- | --- |
|  | df | F | Sig. | df | F | Sig. |
| Time | 9 | 1729.288 | <0.001 | 11 | 22.468 | <0.001 |
| Time*Intensity | 9 | 2.031 | 0.038 | 11 | 3.515 | <0.001 |
| Time*Spectrum | 36 | 84.130 | <0.001 | 11 | 8.863 | <0.001 |
| Time*Intensity *Spectrum | 36 | 3.236 | <0.001 | 11 | 4.026 | <0.001 |
| Error | 180 |  |  | 88 |  |  |

**Table S3.** Three-way analysis of variance for the effects of CO_2_, radiation spectrum, and radiation intensity on PAR- and UVR-driving carbon fixation of *S. costatum.* The asterisk means the interactive effect, df means degree of freedom, F means the value of F statistic, and Sig. means *p*-value.

| Source | PAR-driving carbon fixation | | | UVR-driving carbon fixation | | |
| --- | --- | --- | --- | --- | --- | --- |
|  | df | F | Sig. | df | F | Sig. |
| CO_2_ | 4 | 1.063 | .385 | 4 | 1.808 | 0.152 |
| Spectrum | 5 | 8.854 | .000 | 4 | 3.882 | 0.011 |
| Intensity | 4 | 79.305 | .000 | 4 | 234.650 | <0.001 |
| CO_2_* Spectrum | 8 | 2.432 | .027 | 4 | 1.868 | 0.141 |
| CO_2_*Intensity | 4 | 3.194 | .021 | 4 | .939 | 0.454 |
| Spectrum*Intensity | 8 | 5.919 | .000 | 4 | 7.249 | <0.001 |
| CO_2_*Spectrum* Intensity | 8 | 1.813 | .098 | 4 | 1.603 | 0.198 |
| Error | 48 |  |  | 31 |  |  |

**Table S4.** Three-way analysis of variance for the effects of CO_2_, radiation spectrum, and radiation intensity on PAR- and UVR-driving F'_v_/F'_m_ of *S. costatum.* The asterisk means the interactive effect, df means degree of freedom, F means the value of F statistic, and Sig. means *p*-value.

| Source | PAR-driving F'_v_/F'_m_ | | | UVR-driving F'_v_/F'_m_ | | |
| --- | --- | --- | --- | --- | --- | --- |
|  | df | F | Sig. | df | F | Sig. |
| CO_2_ | 4 | 182.910 | <0.001 | 4 | 48.242 | <0.001 |
| Spectrum | 5 | 9.747 | <0.001 | 4 | 151.147 | <0.001 |
| Intensity | 4 | 771.346 | <0.001 | 4 | 124.133 | <0.001 |
| CO_2_* Spectrum | 8 | 10.880 | <0.001 | 4 | 16.794 | <0.001 |
| CO_2_*Intensity | 4 | 102.737 | <0.001 | 4 | 6.082 | 0.001 |
| Spectrum*Intensity | 8 | 5.729 | <0.001 | 4 | 45.960 | <0.001 |
| CO_2_*Spectrum* Intensity | 8 | 2.508 | 0.023 | 4 | 5.342 | 0.002 |
| Error | 48 |  |  | 31 |  |  |
